# A Water‐Saving Drought Survival Phenotype in a Wheat TILLING Mutant Involves Survival‐Biased Metabolic and Phosphorylation Reprogramming

**DOI:** 10.1111/pce.70546

**Published:** 2026-04-19

**Authors:** Ryosuke Mega, Shun‐ichiro Hirata, Kota Yamashita, Hinano Takase, Taishi Umezawa, Yasuko Watanabe, June‐Sik Kim, Tomoyuki Kosaka, Akihiro Nieda, Hisashi Tsujimoto

**Affiliations:** ^1^ Graduate School of Agricultural Science Kobe University Kobe Japan; ^2^ Graduate School of Sciences and Technology for Innovation Yamaguchi University Yamaguchi Japan; ^3^ Graduate School of Bio‐Applications and Systems Engineering Tokyo University of Agriculture and Technology Koganei Japan; ^4^ Faculty of Agriculture Tokyo University of Agriculture and Technology Fuchu Japan; ^5^ RIKEN Centre for Sustainable Resource Science Yokohama Japan; ^6^ Institute of Plant Science and Resources Okayama University Kurashiki Japan; ^7^ Arid Land Research Centre Tottori University Tottori Japan

**Keywords:** drought, metabolic reprogramming, phosphoproteomic profile, TILLING, trade‐off, water‐saving, wheat (*Triticum aestivum* L.)

## Abstract

Drought tolerance in crops often involves trade‐offs between water conservation, growth and reproduction. Understanding how water‐saving strategies are implemented at physiological and metabolic levels remains critical for improving crop performance under water‐limited conditions. Here, we characterise a wheat TILLING mutant, WS1, that exhibits enhanced survival under drought stress. WS1 showed reduced stomatal conductance and transpiration, together with increased carbon isotope discrimination, consistent with reduced water loss. Despite these traits, WS1 displayed weak stomatal responses to exogenous abscisic acid (ABA), indicating that its water‐saving behaviour is not primarily explained by canonical ABA signalling. Metabolomic analyses revealed substantial accumulation of proline and changes in nitrogen and carbon metabolism, suggesting a shift toward a survival‐oriented metabolic state. Phosphoproteomic profiling further identified distinct phosphorylation patterns associated with energy‐related signalling components, including proteins linked to SnRK1 pathways. These molecular features likely reflect an altered energy‐sensing and regulatory state rather than direct causal drivers of the phenotype. Notably, WS1 exhibited reduced fertility, indicating a trade‐off between survival and reproductive investment. Together, our results suggest that WS1 adopts a water‐saving drought survival phenotype characterised by coordinated physiological and metabolic reprogramming, providing a framework for understanding survival‐oriented drought responses and resource allocation strategies in wheat.

## Summary statement

We identify a wheat TILLING mutant with a water‐saving drought survival phenotype, showing reduced stomatal conductance and improved water‐use efficiency. This phenotype is linked to metabolic and phosphorylation reprogramming not explained by canonical ABA responsiveness, including altered SnRK1 signalling and constitutive proline accumulation.

## 1 Introduction

Climate change is intensifying constraints on crop production through rising temperatures, altered precipitation regimes and more frequent extremes, thereby threatening global food security. Increased evaporative demand and drought episodes are projected to further reduce yields of major cereals (Lobell et al. 2011), and compound hot–dry events during the growing season substantially increase the probability of yield losses in rice, maize, soybean and wheat (Heino et al. 2023). Changes in rainfall distribution also destabilise soil moisture availability and increase production risks across regions (Rosenzweig et al. 2014). In wheat, the vulnerability to water limitation is particularly concerning modelling and multi‐model assessments indicate that warming and drought will increasingly constrain production in major wheat‐growing regions (Asseng et al. 2015), and historical analyses show that extreme weather disasters can markedly reduce global crop output (Lesk et al. 2016). With agricultural water resources projected to become increasingly limiting under future climates (Gupta et al. 2020), there is an urgent need to develop strategies that improve drought survival and water‐use efficiency (WUE) in wheat.

Plants adopt multiple drought‐adaptive strategies, including (i) reduced transpirational water loss through stomatal regulation and (ii) metabolic adjustment via accumulation of compatible solutes and reallocation of carbon and nitrogen resources. Stomatal closure is classically governed by abscisic acid (ABA) signalling, in which ABA binding to PYR/PYL/RCAR receptors inhibits PP2Cs, activates SnRK2s and induces ABA‐responsive transcription (Park et al. 2009; Cutler et al. 2010). However, recent studies highlight additional regulatory layers acting on ABA receptors, including phosphorylation‐dependent changes that can modulate receptor stability and signalling output (Chen et al. 2018; Zhang et al. 2018; Li et al. 2019; Yoshida et al. 2019). The TOR kinase is widely recognised as a central regulator of growth–stress balance and can interface with ABA signalling, including via receptor phosphorylation and reciprocal regulation of TOR activity during stress (Wang et al. 2018; Flavell 2023). These observations underscore that drought responses emerge from coordinated hormonal and post‐translational regulatory networks rather than a single linear pathway.

In parallel with TOR‐linked growth control, the energy‐sensing kinase SnRK1 functions as a major integrator of carbon status and stress signalling (Polge and Thomas 2007; Baena‐González and Sheen 2008; Wang et al. 2020a). SnRK1 has been implicated in both ABA‐dependent and ABA‐independent responses and may coordinate metabolic reprogramming under fluctuating resource availability (Jossier et al. 2009; Carianopol et al. 2020; Belda‐Palazón et al. 2022; Zhao et al. 2026). In Arabidopsis, SnRK1‐associated phosphorylation events have been linked to ABA‐independent stress‐responsive transcription through components such as OXS3 and trehalose‐pathway enzymes (e.g. TPS5) (Wurzinger et al. 2018; Xiao et al. 2021). Nevertheless, how SnRK1‐related signalling states relate to water‐saving strategies and drought survival in wheat remains insufficiently defined.

Metabolic adjustment is a hallmark of drought adaptation. Proline (Pro) is a widely conserved compatible solute that contributes to osmotic adjustment and redox homoeostasis, and its accumulation can also be associated with broader changes in growth and stress physiology (Alvarez et al. 2022). Because SnRK1 is a key regulator of energy‐dependent metabolic reprogramming, an SnRK1‐linked regulatory state could plausibly coincide with proline‐associated metabolic configurations under water limitation. However, the relationships among phosphorylation dynamics, energy‐sensing networks and proline metabolism remain incompletely resolved in crop species.

To better understand survival‐oriented drought responses in wheat, we exploited an EMS‐induced TILLING resource in hexaploid wheat that includes mutations in ABA receptor genes (Krasileva et al. 2017). Using a screening strategy based on seedling traits and carbon isotope discrimination, we identified a candidate water‐saving line, Cadenza0773 (WS1), which exhibits enhanced drought survival, reduced stomatal conductance and improved WUE relative to the parental line Cadenza0000 (C). WS1 also shows constitutively elevated expression of TaP5CS1 and increased proline accumulation under non‐stress conditions. Here, we integrate physiological, transcriptomic and phosphoproteomic analyses to characterise the water‐saving drought survival phenotype of WS1 and to define associated metabolic and phosphorylation reprogramming. Rather than asserting a single causal driver, we use these datasets to establish an integrated framework for drought survival–oriented water‐use strategies in wheat.

## 2 Materials and Methods

### 2.1 Plant Materials

The seeds of 129 lines from the hexaploid bread wheat (cv. Cadenza) Targeting Induced Local Lesions in Genomes (TILLING) population used in this study were provided by SeedStor2 (https://www.seedstor.ac.uk/index.php), created by the Germplasm Resources Unit at the John Innes Centre, UK (Rakszegi et al. 2010; Krasileva et al. 2017). Among these, Cadenza0773 (WS1), selected as a candidate water‐saving and drought‐tolerant line based on its sensitivity to abscisic acid (ABA) during seedling elongation and carbon isotope ratio, and the standard line Cadenza0000 (C) were used for the experiments.

### 2.2 Seedling Drought Tolerance Test

C and WS1 seeds were imbibed on Petri dishes and incubated for 2 days in a growth chamber (LH‐411PFDT‐S, Nippon Medical & Chemical Instruments Co. Ltd., Osaka, Japan). Five seedlings were then transplanted into 7.5‐cm pots containing 85 g of soil and grown under well‐watered conditions on trays for 3 weeks in 22°C/17°C of 16 h day/8 h night. Drought stress was imposed by draining water from the trays for 20 days, after which the plants were rewatered, and survival rates were recorded 2 weeks later. The experiment was conducted with five replicates per line.

### 2.3 SPAD Value Measurement

Using the same plants described above, SPAD values were measured over a 2‐week period during the drought treatment. A chlorophyll metre (SPAD‐502Plus, Konica Minolta, Tokyo, Japan) was used to continuously measure the SPAD value at the centre of the fully expanded youngest leaf at the third week of cultivation. The standardised SPAD value was calculated by setting the SPAD value on the first day of drought treatment to 1 and dividing the SPAD value of each subsequent measurement day by that initial value.

### 2.4 Water Utilisation Measurement

C and WS1 seeds were germinated on Petri dishes, and six seedlings were transplanted into 8‐cm pots and grown in a growth chamber for 1 week. The seedlings were then transferred to containers and watered until the total weight reached approximately 5000 g. After a certain period, the containers were weighed again and rewatered to restore the total weight to about 5000 g. This process was repeated until the plants withered. For the main experiment, seeds were germinated as in the drought test and transplanted into 7.5‐cm pots, followed by 1 week of growth in a controlled‐environment chamber. One‐week‐old seedlings were then transplanted into 7‐L containers (342 × 193 × 169 mm; JEJ Astage Co. Ltd., Niigata, Japan; Cat. No. 4991068143951), six plants per container, and grown under 16 h day/8 h night. After each irrigation, the total container weight was recorded, and by remeasuring the weight after a set period, water use during that interval was determined. This procedure was repeated throughout the cultivation period to calculate total water use. Aluminium foil was placed on the soil surface to minimise water loss through evaporation. After cultivation, the plants were dried, and their biomass was measured. The experiment was conducted with three replicates per line.

### 2.5 ^13^C Analysis

The carbon isotope composition (δ^13^C) of wheat leaves was analysed using an elemental analyser interfaced with a continuous‐flow isotope ratio mass spectrometer (EA/IRMS; Thermo Fisher Scientific). The samples to be analysed were prepared from flag leaves of matured plants after Water Utilisation Measurement. One mg of flag leaf tissues was filled into tin capsules (5 × 9 mm, LUDI Swiss). Each sample was measured against standard CO_2_ calibrated with an isotope standard (accuracy of calibration ±0.066‰ SD). ^13^C discrimination was calculated as

$$\delta^{13}C=\left(R_{\text{sample}}/R_{\text{standard}}-1\right)\times 1000,$$

where R represents the ^13^C/^12^C isotope ratio of samples and standards.

### 2.6 Photosynthesis Measurement

Seeds were germinated as described in the drought tolerance test and transplanted into large pots, followed by 1 week of growth in a controlled‐environment chamber. The pots were grown under blue‐red light conditions in 23°C. Photosynthetic parameters were measured on the fully expanded youngest leaves of 3–4‐week‐old wheat plants using a portable photosynthesis system (LI‐6800, LI‐COR, Nebraska, USA). Light‐dependent photosynthesis was measured at a CO₂ concentration of 400 ppm under photosynthetic photon flux density (PPFD) of 0, 20, 50, 100, 200, 500, 1000, 1500, 2000, and 3000 µmol m⁻² s⁻¹. CO₂‐dependent photosynthesis was measured under a constant light intensity of 1500 µmol m⁻² s⁻¹ with CO₂ concentrations of 0, 50, 100, 200, 400, 500, 800, 1000, 1500, and 2000 ppm. Water use efficiency (WUE) was calculated by dividing the CO₂ assimilation rate (µmol CO₂ m⁻² s⁻¹) by the transpiration rate (mmol H₂O m⁻² s⁻¹). Measurements were performed with six replicates per line. A CO₂ gas cylinder was used as the CO₂ source.

### 2.7 Stomatal Density Measurement

The stomata on the flag leaves of plants grown in the water requirement experiment were photographed at a resolution of 2592 × 1944 pixels using a portable stomatal imaging device (Phytometrics Co. Ltd., Shizuoka, Japan). The total number of stomata in each image was counted. An objective micrometre was photographed at the same magnification as the leaf images, and the total image area was calculated using ImageJ. Stomatal density was then determined by dividing the total number of stomata by the image area. Measurements were performed with 18 replicates per line.

### 2.8 Fertility Rate Assessment

Using the dried spikes of plants from the utilisation measurement, the total number of florets and fertile florets in each spikelet were examined to calculate the ratio: fertile/total.

### 2.9 RNA Extraction

Ten‐day‐old wheat seedlings were sprayed with 15 mL of either mock solution (0.05% Tween 20 + DMSO) or ABA solution (0.05% Tween 20 + 50 µM ABA). Samples were collected 24 h after treatment, immediately frozen in liquid nitrogen, and stored at −80°C. Total RNA was extracted following the manufacturer's instructions for TRIzol Reagent (Thermo Fisher Scientific, Massachusetts, USA; Cat. No. 15596026). Approximately 50 mg of frozen, ground tissue was mixed with 500 µL of TRIzol Reagent and vortexed for 30 s, followed by the addition of 100 µL of chloroform and vortexing for 15 s. After incubation at room temperature for 1 min, samples were centrifuged at 12 000*g* for 15 min at 4°C. The aqueous phase (100 µL) was transferred to a new tube, mixed with 250 µL of isopropanol, incubated for 10 min at room temperature, and centrifuged again at 12 000*g* for 10 min at 4°C. The supernatant was discarded, and the pellet was washed twice with 500 µL of 75% RNase‐free ethanol, vortexed, and centrifuged (12 000*g*, 5 min, 4°C). After removing the ethanol, the pellet was air‐dried for 10 min at room temperature, then dissolved in 50 µL of RNase‐free water and incubated at 65°C for 10 min. RNA quality was confirmed by 0.8% agarose gel electrophoresis, and concentration and purity were determined using a spectrophotometer (Colibri +, Berthold, Japan), ensuring an A260/A280 ratio greater than 1.8. Samples were stored at −20°C.

### 2.10 qRT‐PCR

For reverse transcription, ReverTra Ace qPCR RT Master Mix with gDNA Remover (Toyobo Co. Ltd., Osaka, Japan; Cat. No. FSQ‐301) was used. RNA samples equivalent to 500 ng were mixed with 2 µL of 4× DNA Master Mix and adjusted to a total volume of 8 µL with RNase‐free water. The mixture was incubated at 37°C for 5 min, followed by the addition of 2 µL of 5× RT Master Mix II, and the reverse transcription reaction was performed using a thermal cycler (Gene Atlas G02, Astec Co. Ltd., Fukuoka, Japan) under the following conditions: 37°C for 15 min, 50°C for 5 min, and 98°C for 5 min. Real‐time PCR was performed using THUNDERBIRD Next SYBR qPCR Mix (Toyobo; Cat. No. QPX‐201). The reaction mixture (total 20 µL) contained 5 µL of cDNA, 10 µL of THUNDERBIRD Next SYBR qPCR Mix, 1 µL each of forward and reverse primers (6 pmol/µL), and 3 µL of ultrapure water. The mixture was loaded into PCR tubes (BIO‐RAD, California, USA, Cat. No. TLS0851). Reactions were run on a CFX Opus 96 Real‐Time PCR System (BIO‐RAD) at Yamaguchi University under the following conditions: 94°C for 60 s (1 cycle), followed by 40 cycles of 65°C for 15 s, 65°C for 15 s, and 72°C for 60 s. To perform absolute quantification of gene expression, serial dilutions of plasmids containing the target gene fragments were prepared. Plasmids were constructed by cloning PCR‐amplified fragments of the target regions, which correspond to the primer pairs used for real‐time PCR. Using cDNA from standard line C as a template, standard PCR amplification was performed with 10 µL of 2× PCR Master Mix (Nippon Genetics Co. Ltd., Tokyo, Cat. No. FG‐MM320), 1 µL of cDNA, 0.4 µL each of forward and reverse primers (10 pmol/µL), and 8.2 µL of ultrapure water. The PCR programme was as follows: 94°C for 2 min (1 cycle); 98°C for 10 s, 64°C for 30 s, 72°C for 20 s (5 cycles); 98°C for 10 s, 61°C for 30 s, 72°C for 20 s (5 cycles); 98°C for 10 s, 58°C for 30 s, 72°C for 20 s (30 cycles); and 72°C for 7 min (1 cycle). For TA cloning, 0.5 µL of T‐Vector pMD20 (Takara Bio Inc., Shiga, Japan; Cat. No. 3270), 0.5 µL of PCR product, and 1.5 µL of ultrapure water were mixed in a 1.5 mL tube, followed by the addition of 2.5 µL of DNA Ligation Mix <Mighty Mix> (Takara Bio; Cat. No. 6023) and incubation at 16°C for 30 min. The entire ligation mixture was transformed into 50 µL of DH5α competent cells (Takara Bio, Cat. No. 9057) and spread on LB agar plates containing 100 µg/mL ampicillin. For blue‐white screening, IPTG and X‐Gal were added to the plates according to manufacturer's protocol. White colonies were selected, suspended in 1 mL of LB liquid medium (+ 100 µg/mL ampicillin), and cultured overnight at 37°C. Plasmids were purified using a Plasmid Miniprep Purification Kit (GeneMark, Atlanta, USA, Cat. No. DP01‐300). The purified plasmid DNA concentration was measured with a spectrophotometer, and the copy number was calculated based on the concentration. A standard curve was generated for the pMD20 plasmid containing the target DNA sequence to determine absolute gene expression levels. The primers listed in Supporting Information Table S1 were used for each gene.

### 2.11 ABA Quantification

From the fully expanded youngest leaves of 34‐day‐old plants used for photosynthetic activity measurements, five leaf discs were collected using a leaf punch (Kai Industries Co. Ltd., Gifu, Japan; BPP‐30F) and immersed in 100 µL of 80% methanol, then stored at −20°C. Three weeks after sampling, 50 µL of the extract was mixed with 40 µL of 20% methanol and 10 µL of internal standard solution (0.1 ppm d₆‐ABA). The mixture was transferred to a Nanosep MF 0.45 µm filter (Pall Corporation, New York, USA, Cat. No. ODPTFE04C35) and centrifuged at 14,000 × g for 5 min at room temperature. The filtrate was transferred to vials (Technolab SC Co. Ltd., Osaka, Japan, Cat. No. ML345‐3012) for LC–MS analysis. LC–MS analysis was conducted using the 3200 QTRAP LC/MS/MS System (AB Sciex, Tokyo, Japan). For LCMS, Mightysil RP‐18 GPⅡ column (150 × 2.0 mm, 5 µm; Kanto Chemical Co. Inc., Tokyo, Japan; Cat. No. 25697‐96) was used. The mobile phase consisted of solvent A (0.1% formic acid in H₂O) and solvent B (0.1% formic acid in acetonitrile). The gradient programme was as follows: 0–5 min, 20% B; 5–20 min, 90% B; 20–30 min, 90% B; 30–35 min, 20% B. The flow rate was 0.2 mL min⁻¹, the injection volume was 4.0 µL, and the column temperature was maintained at 40°C. For ABA, the mass transition was m/z 263 (Q1) for precursor ion to 153 (Q3) for product ion with declustering potential (DP) = −50 V, entrance potential (EP) = − 11 V, and collision energy (CE) = −18 V. For d₆‐ABA, the mass transition was m/z 269 (Q1) to 159 (Q3) with DP = −45 V, EP = −9.5 V and CE = −12 V.

### 2.12 Phosphoproteome Analysis

Protein extraction and digestion were performed according to a previous study with some modifications (Yamashita et al. 2025). In brief, frozen samples (10 mg) from the mock‐treated plants of each line were dispensed into antistatic tubes, and 100 µL of protein extraction buffer (6 M guanidine hydrochloride [FUJIFILM Wako Pure Chemical Corp., Cat. No. 072‐05001] +100 mM Tris‐HCl, pH 9.0) was added. The mixture was vortexed and heated at 100°C for 5 min. After cooling on ice, ultrasonication was performed for 20 s × 3 cycles, followed by centrifugation (13 200 rpm, 20 min, 4°C). The supernatant was transferred to ProteoSave SS Microtubes (Sumitomo Bakelite Co. Ltd., Akita, Japan, Cat. No. MS‐4215M). Subsequently, 400 µL of methanol was added and vortexed, followed by the addition of 100 µL of chloroform and mixing. Then, 300 µL of ultrapure water was added and vortexed again. After centrifugation (13 200 rpm, 3 min, 4°C), the upper aqueous layer was removed, 400 µL of methanol was added, mixed, and centrifuged again (13 200 rpm, 3 min, 4°C). The liquid was completely removed, and the protein pellet was air‐dried and stored at − 80°C. The protein pellets were resuspended in a digestion buffer containing 100 mM Tris‐HCl (pH 9.0), 12 mM SLS, and 12 mM SDC. 400 µg of protein per sample was incubated in a reduction buffer [10 mM DTT, 100 mM ammonium bicarbonate] for 30 min at 24°C, followed by alkylation buffer [40 mM CAA, 100 mM ammonium bicarbonate] for 20 min at 24C in the dark. After a five‐fold dilution with 100 mM ammonium bicarbonate, proteins were digested overnight at 37°C with 4 µg trypsin (Promega). Phase transfer surfactants (such as SLS, SDC) were removed according to a previous study (Yamashita et al. 2025). After removal, 10% of the total volume of peptide samples was set aside for global proteomic analysis, and phosphopeptides were enriched using hydroxy acid‐modified metal oxide chromatography (HAMMOC) as described in a previous study (Sugiyama et al. 2007 ). Digested peptides or enriched phosphopeptides were desalted using an in‐house Stage‐tip made with SDB Empore discs (CDS). After desalting, the phosphopeptides were dried and stored at −80°C until LC‐MS/MS analysis.

### 2.13 LC‐MS/MS Analysis and Raw Data Processing

LC–MS/MS analysis was performed using an Easy‐nLC 1200 system coupled to an Orbitrap Exploris 480 mass spectrometer equipped with a FAIMS Pro interface (Thermo Fisher Scientific). The dried peptides were dissolved in 10 µL of 0.1% formic acid + 2% acetonitrile solution, and peptide concentration was measured. Approximately 750 ng of peptides were loaded directly into a C18 nano HPLC capillary column (NTCC‐360/75‐3, 75 µm ID × 15 cm L, Nikkyo Technos) and separated using a 120‐min nonlinear gradient. Mass spectrometry was performed using data‐dependent acquisition (DDA) with FAIMS compensation voltages of −40 and −60 V. Data analysis was conducted using Proteome Discoverer 2.5 (Thermo Fisher Scientific) with the SEQUEST search engine against the *Triticum aestivum* RefSeq v1.1 database. SEQUEST parameters were as follows: Digestion enzyme, Trypsin; Maximum missed cleavages, 2; Peptide length, 6–144; Precursor mass tolerance, 10 ppm; Fragment mass tolerance, 0.02 Da; Static modification, Carbamidomethylation (C); Variable modifications, Oxidation (M), N‐terminal acetylation, and phosphorylation (S, T, Y); Maximum variable modifications per peptide, 3. Both peptide and protein identifications were filtered at a False Discovery Rate (FDR) of < 1%. Phosphorylation site localisation probabilities were calculated using IMP‐ptmRS, with a threshold set at ≥ 75%.

### 2.14 Mutated Gene Analysis of TILLING

Mutation information for WS1 was obtained from the TILLING database (https://dubcovskylab.ucdavis.edu/wheat-tilling). The Gene IDs from the mutation data were converted to the corresponding Gene IDs in *Triticum aestivum* RefSeq v1.1. The converted Gene IDs were then used for gene searches using the BLAST tools available at EnsemblPlants (https://plants.ensembl.org/Triticum_aestivum/Info/Index) and TAIR (https://www.arabidopsis.org/).

### 2.15 Transcriptome Analysis

NGS library preparation and sequencing procedures from leaf RNA samples were conducted as described previously (Kim et al. 2025). In detail, mRNA was isolated from total RNA samples using NEXTFLEX Poly(A) Beads 2.0 (PerkinElmer), and sequencing libraries were prepared using the NEXTFLEX Rapid Directional RNA‐Seq Kit 2.0 (PerkinElmer). Quality control and quantification of the prepared libraries were performed using a TapeStation system (Agilent Technologies). The libraries were sequenced in paired‐end read (2 × 150 nucleotides) mode using a DNBSEQ‐G400 system (MGI Tech). The resulting reads were quality‐filtered using Trimmomatic‐v0.39 with default parameters (Bolger et al. 2014). The cleaned reads were aligned to the transcripts of the wheat reference genome RefSeq‐v2.1 using the selective alignment method of Salmon‐v1.10.0 with default parameters (Zhu et al. 2021; Srivastava et al. 2020). Differentially expressed genes were identified using DESeq. 2‐v1.50.2 and tximport‐v1.38.2 in R‐v4.5.2 (Love et al. 2014; Soneson et al. 2015; R Core Team 2023). Gene set enrichment analysis was conducted using clusterProfiler‐v4.18.4, with GO and KEGG databases retrieved from Ensembl Plants‐r60 (Cunningham et al. 2022).

## 3 Results

### 3.1 WS1 Exhibits a Water‐Saving Drought Survival Phenotype

**Figure 1:**
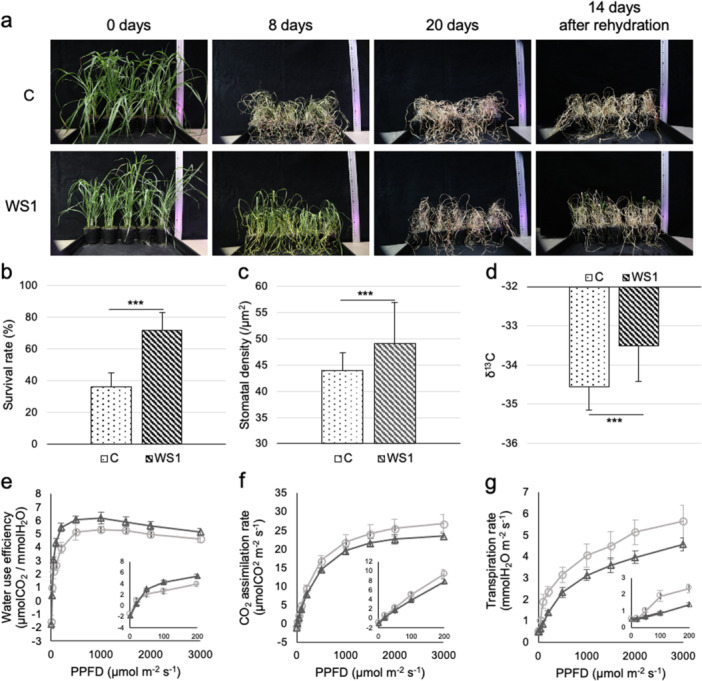
Physiological and photosynthetic characteristics of the drought‐tolerant wheat line WS1. (a) Control line (C) and drought‐tolerant line WS1 subjected to progressive water‐withholding treatment. Representative images are shown at 0, 8 and 20 days after the onset of water withholding, and after 14 days of rehydration. (b) Survival rate of C and WS1 plants following drought stress and subsequent rehydration. (c) Stomatal density measured on the abaxial leaf surface of fully expanded leaves. (d) Carbon isotope composition (δ¹³C) of leaf tissues, indicating long‐term intrinsic water‐use efficiency. (e) Light‐response curves of instantaneous water‐use efficiency (WUE), calculated as the ratio of CO₂ assimilation rate to transpiration rate, plotted against photosynthetic photon flux density (PPFD). (f) Light‐response curves of CO₂ assimilation rate as a function of PPFD. (g) Light‐response curves of transpiration rate as a function of PPFD. Data are presented as means ± SD. Asterisks indicate statistically significant differences between C and WS1 (*p < 0.05, **p < 0.01, ***p < 0.001, Student's *t*‐test).

To identify wheat lines with enhanced drought adaptation, we screened a TILLING population comprising 1,200 hexaploid wheat lines carrying mutations in abscisic acid (ABA) receptor genes (TaPYLs) based on Mega et al. 2019. From 127 selected mutant lines, screening based on seedling elongation assays and carbon isotope discrimination (δ¹³C) values of flag leaves grown under field conditions identified one line with the highest δ¹³C value, which we designated Water Saving 1 (WS1) (Supporting Information Figures S1, S2). To assess drought performance, a seedling‐based drought survival assay was conducted. Under water‐deficit conditions, WS1 displayed delayed chlorosis and significantly higher survival rates after rewatering compared with the standard line C (Figure 1a,b, Supporting Information Figure S3a), demonstrating enhanced drought survival capacity. Physiological measurements revealed that stomatal density in WS1 was significantly higher than that in C (Figure 1c, Supporting Information Figure S3c,d). The δ¹³C values were significantly higher in WS1 (Figure 1d), reflecting improved intrinsic water‐use efficiency (WUE) (Farquhar et al. 1989). Gas‐exchange analyses showed that both transpiration rate and CO₂ assimilation rate were reduced in WS1 relative to C, indicating a constitutively reduced stomatal aperture. However, the reduction in CO₂ assimilation in WS1 was proportionally smaller than that of transpiration, resulting in an approximately 15% increase in WUE (Figure 1e–g). The analysis of A–Ci curves revealed no significant differences between WS1 and C (Supporting Information Figure S3b), indicating that the enhanced WUE in WS1 is not attributable to the altered Rubisco activity. Together, these results indicated that WS1 adopts a water‐saving drought survival phenotype, characterised by reduced water loss through stomatal regulation while maintaining relatively efficient carbon assimilation.

### 3.2 ABA Signalling Contributes Only Marginally to Drought‐Related Traits in WS1

**Figure 2:**
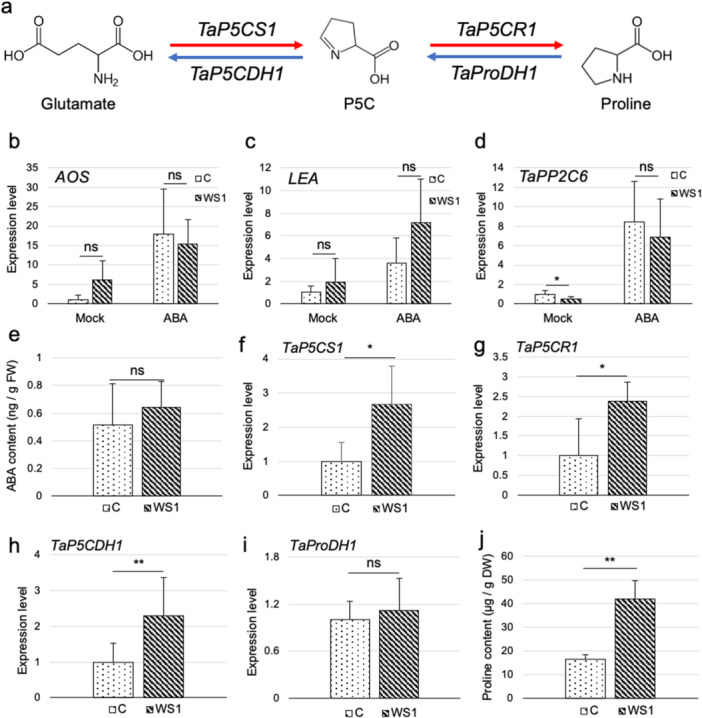
Enhanced proline accumulation in WS1 is associated with altered expression of proline metabolic genes independently of ABA accumulation. (a–d) Relative expression levels of ABA‐responsive marker genes AOS (a), LEA (b), TaPP2C6 (c) and TaP5CS1 (d) in control line (C) and WS1 under mock or ABA‐treated conditions. (e) Endogenous ABA content in leaves of C and WS1 plants under mock conditions. (f) Proline content in leaves of C and WS1 plants. Gene expression levels were normalised to an internal reference gene and are shown relative to the control line. (g) Schematic representation of the proline biosynthesis and catabolic pathway in wheat. Glutamate is converted to Δ¹‐pyrroline‐5‐carboxylate (P5C) by TaP5CS1, followed by reduction to proline by TaP5CR1. Proline catabolism is mediated by TaProDH1 and TaP5CDH1. (h–j) Relative expression levels of proline metabolism–related genes TaP5CR1 (h), TaP5CDH1 (i), and TaProDH1 (j) under mock conditions. Data are presented as means ± SD (*n* = 5 at all). Asterisks indicate statistically significant differences between C and WS1 (*p < 0.05, **p < 0.01, ***p < 0.001, Student's *t*‐test; ns, not significant). [Color figure can be viewed at wileyonlinelibrary.com]

Because ABA signalling is a central regulator of drought responses, we evaluated the ABA responsiveness of WS1. Exogenous ABA application to 1‐week‐old seedlings resulted in comparable reductions in shoot elongation rates in both WS1 and C relative to their respective mock treatments (Supporting Information Figure S1), indicating similar sensitivity to ABA at the physiological level. Consistently, transcript levels of ABA‐responsive genes, including AOS and LEA, did not differ significantly between WS1 and C under either mock or ABA‐treated conditions (Figure 2a,b). Although TaPP2C6 expression was higher in C under mock conditions, no difference was observed following ABA treatment (Figure 2c). Notably, the expression of TaP5CS1, encoding a key enzyme in proline biosynthesis, was constitutively higher in WS1 than in C regardless of ABA treatment (Figure 2d). Measurement of endogenous ABA content under non‐stress conditions revealed no significant difference between WS1 and C (Figure 2e), indicating that altered ABA accumulation does not account for the WS1 phenotype. Collectively, these results suggest that canonical ABA signalling is not substantially altered in WS1, and that drought‐related traits are likely mediated by regulatory mechanisms which cannot be solely attributed to ABA signalling.

### 3.3 Metabolic Reprogramming Supports Drought Survival in WS1

**Figure 3:**
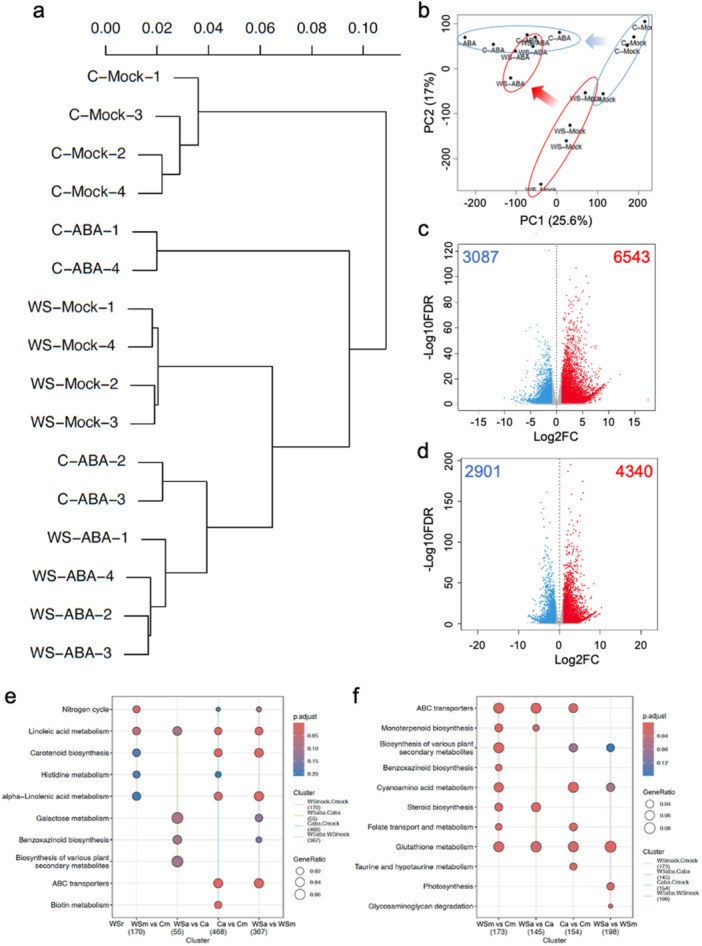
Transcriptomic signatures indicate constitutive activation of stress‐related pathways in WS1. (a) Dendrogram of RNA‐seq samples based on their transcriptome‐wide Spearman correlation. Quadruplicated control (C) and WS1 (WS) samples are shown under mock or ABA treatment conditions. (b) Principal component analysis (PCA) of transcriptomic profiles. Percentages indicate the variance explained by each principal component. (c, d) Volcano plots showing differentially expressed genes (DEGs) between WS1 and C under mock (c) and ABA‐treated (d) conditions. Numbers indicate up‐ and down‐regulated genes passing the significance threshold (|fold‐change | > = 2 and false discovery rate (FDR) < 0.05). (e, f) KEGG pathway enrichment analysis of upregulated (e) and downregulated (f) DEGs identified in each comparison. Dot size represents the gene ratio (proportion of DEGs in each pathway), and colour indicates FDR. [Color figure can be viewed at wileyonlinelibrary.com]

Given the constitutive upregulation of TaP5CS1, we further examined proline metabolism in WS1. LC–MS analysis revealed approximately two‐fold higher proline accumulation in WS1 compared with C (Figure 2f). Consistent with the proline content difference, under non‐stress conditions, expression of TaP5CR1 and TaP5CDH1 was significantly higher in WS1 than in C, whereas TaProDH1 expression remained unchanged (Figure 2g–i). Transcriptome analysis further revealed broad metabolic reprogramming in WS1. Hierarchical clustering and principal component analysis showed that mock‐treated WS1 samples clustered closely with ABA‐treated samples of both genotypes (Figure 3a,b), suggesting a constitutively activated stress‐related transcriptional state. Related to this point, we compared the volcano plots of C and WS1 between in the presence and absence of ABA (Figure 3c,d), indicating WS1 was not sensitive to ABA treatment. KEGG pathway enrichment analysis of genes upregulated in WS1 under mock conditions identified significant enrichment of pathways related to linoleic acid metabolism, carotenoid biosynthesis, histidine metabolism, and nitrogen cycling (Figure 3e). Conversely, genes downregulated in WS1 were enriched in pathways associated with secondary metabolism, transport processes, and reproductive development (Figure 3f), indicating a reallocation of metabolic resources away from growth‐related processes. Notably, genes involved in asparagine metabolism were consistently upregulated in WS1 under both mock and ABA‐treated conditions (Supporting Information Figure S4a). Given previous reports linking asparagine accumulation to stomatal regulation in drought and dark responses (Lea et al. 2007; Itam et al. 2020), these findings suggest that nitrogen‐related metabolic adjustment contributes to the water‐saving phenotype in WS1.

### 3.4 Distinct Phosphorylation Profiles Are Associated With Energy‐Related Signalling in WS1

**Figure 4:**
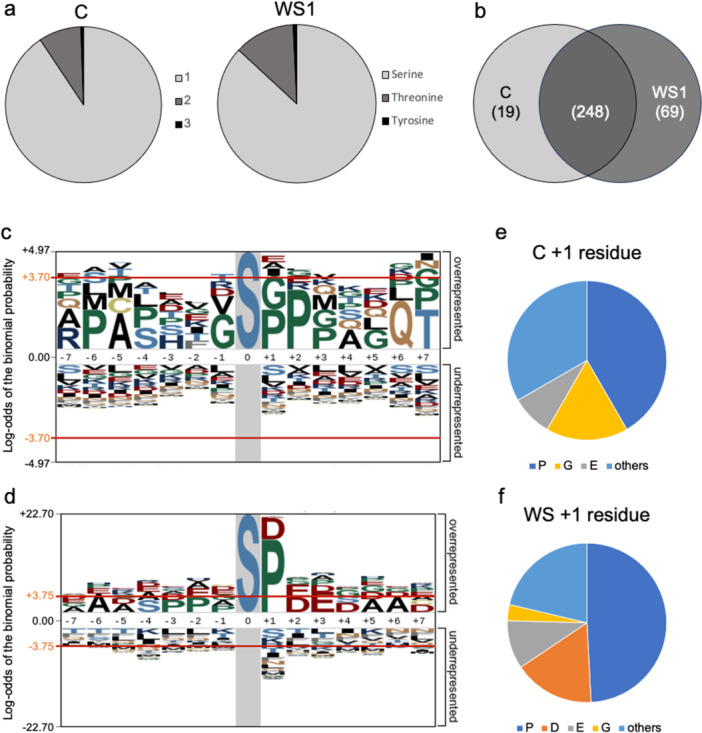
Global phosphoproteomic analysis reveals altered phosphorylation motifs in WS1. (a) Distribution of phosphorylated amino acid residues (Ser, Thr, Tyr) identified in C and WS1. (b) Venn diagram showing overlap of phosphopeptides detected in C and WS1. (c, d) Motif analysis of phosphorylation sites in C (c) and WS1 (d). The panels in (c) and (d) were drawn by using pLogo (O'Shea et al. 2013). Sequence logos represent over‐ and under‐represented amino acids surrounding the phosphorylated residue. (e, f) Frequency of amino acids at the +1 position relative to the phosphorylation site in C and WS1, highlighting differences in motif preference. [Color figure can be viewed at wileyonlinelibrary.com]

**Figure 5:**
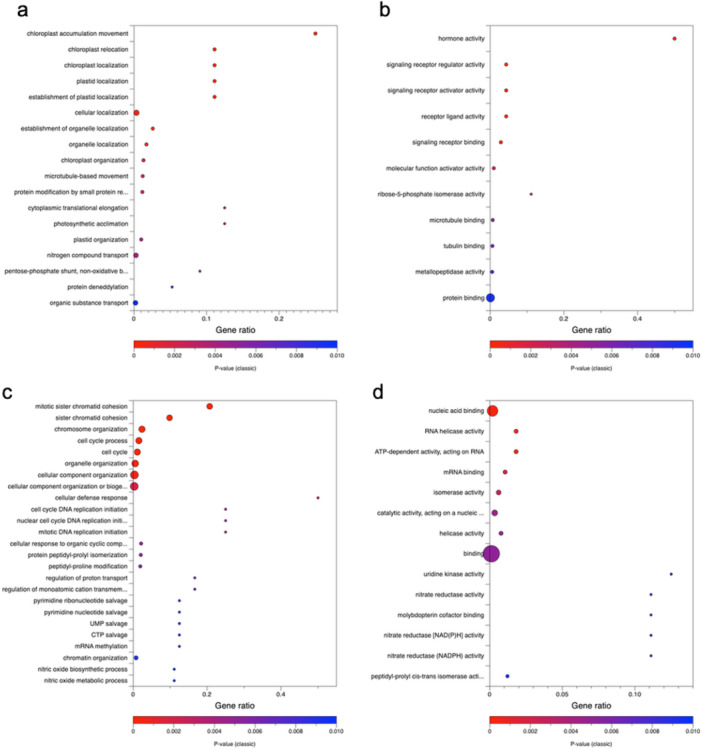
Functional enrichment analysis of differentially phosphorylated proteins. (a–d) Gene Ontology (GO) enrichment analysis of differentially phosphorylated proteins between C and WS1. Biological process (a, c) and molecular function (b, d) categories are shown for each comparison. Dot size indicates gene ratio, and colour represents enrichment significance (*p* value). [Color figure can be viewed at wileyonlinelibrary.com]

To explore post‐translational regulation associated with the WS1 phenotype, we conducted phosphoproteomic analysis. A total of 2711 phosphorylated peptides were identified, predominantly containing serine residues (Figure 4a). Among these, 69 and 19 peptides were uniquely phosphorylated in WS1 and C, respectively (Figure 4b and Supporting Information Tables S2–5). Phosphorylation of the C‐terminal region of TaPYL2A, including the serine residue altered by a missense mutation in WS1, was not detected in either genotype (Supporting Information Figure S5), suggesting that this mutation might not directly affect detectable phosphorylation patterns or the mass spectrum of the related phosphopeptides might not be detected. Motif analysis revealed distinct enrichment of acidic and proline‐containing motifs surrounding phosphorylated residues in WS1, whereas no significant motif enrichment was observed in C (Figure 4c,d). Likewise, the previous phosphoproteomic study in bread wheat also detected proline and aspartic acid residues at the +1 position of the phosphorylated serine in the drought tolerant cultivar (Zhang et al. 2014). Gene Ontology enrichment analysis showed that phosphorylated proteins in WS1 were associated with nuclear functions, RNA binding, and cell cycle regulation, whereas those in C were enriched for chloroplast‐related and hormone‐associated processes (Figure 5a–d). Collectively, these data indicate that WS1 exhibits a distinct phosphorylation landscape associated with altered energy‐related and regulatory processes, consistent with a survival‐oriented physiological state.

### 3.5 Reduced Fertility Reflects a Trade‐Off Associated With Drought Survival

Although WS1 exhibited reduced water use throughout the growth period (Supporting Information Figure S6c), it also showed a significant reduction in yield‐related traits under controlled growth conditions (Supporting Information Figure S6d–h). Yield per unit water was lower in WS1, whereas biomass produced per unit water did not differ significantly between WS1 and C (Supporting Information Figure S6i,j). Morphological analyses indicated that the number of florets per spike was similar between genotypes, whereas grain number per spike was markedly reduced in WS1 (Supporting Information Figure S6e), identifying reduced fertility as the primary cause of yield loss (Supporting Information Figure S6k). Consistently, transcriptomic data revealed downregulation of genes associated with pollen recognition and reproductive processes in WS1 (Supporting Information Figure S4b). These findings indicate that reduced fertility in WS1 represents an intrinsic trade‐off associated with a survival‐oriented, water‐saving phenotype, rather than a secondary consequence of drought stress per se.

## 4 Discussion

### 4.1 WS1 Accepts the Yield Penalty to Gain a Water‐Saving Drought Survival Phenotype

**Figure 6:**
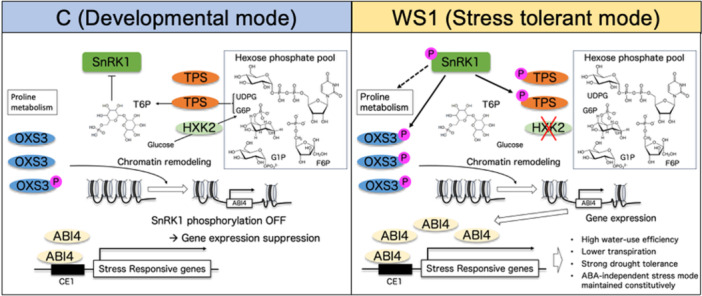
Hypothetical model of a water‐saving drought survival phenotype in the wheat TILLING mutant WS1. Proposed hypothetical model illustrating differences between the developmental growth mode of the control line (C) and the stress‐tolerant mode of WS1. In the control line (C), SnRK1‐associated signalling is relatively inactive, with limited phosphorylation of downstream components such as OXS3 and TPS5. Hexose phosphate pools (e.g. G6P and related metabolites) are maintained at levels consistent with growth‐oriented metabolism, chromatin remains in a relatively closed state, and expression of stress‐responsive genes is suppressed, resulting in a developmental growth mode. In contrast, WS1 exhibits a distinct phosphorylation pattern characterised by enhanced phosphorylation of SnRK1‐associated components, including OXS3 and TPS5. This state is accompanied by altered control of the hexose phosphate pool, activation of proline metabolism, and chromatin remodelling that favours transcription of stress‐responsive genes, partly mediated by ABI4. Together, these molecular features are consistent with constitutive maintenance of a stress‐associated regulatory state in WS1, supporting reduced transpiration, higher water‐use efficiency, and enhanced drought survival. Notably, this stress‐tolerant mode is proposed to operate largely independently of strong canonical ABA activation under non‐stress conditions. [Color figure can be viewed at wileyonlinelibrary.com]

The drought‐tolerant wheat line WS1, identified from a TILLING population, exhibits slightly smaller than the standard line C as shown in Supporting Information Figure S6. The value of WS1 in biomass and survival rate was 0.81‐fold as low as and twofold as high as that of C. Indeed, the survival rate normalised by biomass of WS1 was clearly higher than that of C (Figure 6l), indicating WS1 is truly more drought‐tolerant than C. This difference cannot be fully explained by reduced plant size, as indicated by biomass‐normalised survival rates. Instead, WS1 must accept the yield penalty. Actually, yield‐related traits (Yield, Grain per spike and Spike number) other than Biomass of WS1 were lower than those of C (Figure 6d–f). Although instantaneous WUE was increased (Figure 1e), biomass‐based WUE did not differ, indicating that the apparent water‐saving phenotype is partly associated with reduced growth rather than improved carbon gain efficiency. This suggests that the yield penalty of WS1 is more severe during seed development than vegetative stage due to related mutations. Additionally, the lower fertility of WS1 also supports it (Supporting Information Figure S6k). These phenotypes in WS1 reveal a fundamental trade‐off between plant survival and reproductive allocation. Thus, this type of trade‐off needs to be solved. Genetic improvement as facilitating seed development is essential in leveraging breeding.

### 4.2 Stress‐Associated Traits in WS1 Are Largely Independent of Canonical ABA Signalling

WS1 also exhibits a characteristic combination of physiological and molecular traits even under non‐stress conditions. Transcriptomic profiling showed that mock‐treated WS1 seedlings clustered closely with ABA‐treated samples of both WS1 and the standard line C, suggesting that WS1 maintains a stress‐associated transcriptional state in the absence of exogenous ABA. However, physiological assays and gene expression analyses indicated that canonical ABA responsiveness is not markedly altered in WS1 (Figure 2b–d). Together with the lack of differences in endogenous ABA content and the expression of representative ABA‐responsive genes, these results suggest that WS1 expresses its water‐saving phenotype largely through regulatory routes not via canonical ABA signalling, rather than through constitutively strengthened one.

A prominent ABA‐independent like feature of WS1 is the constitutively high expression of TaP5CS1, a key gene in proline biosynthesis, accompanied by increased proline accumulation under non‐stress conditions. Proline is widely implicated in drought adaptation as an osmoprotectant and as a broader regulator of cellular homoeostasis (Alvarez et al. 2022). Importantly, none of the analysed proline‐metabolism genes carried mutations in WS1, implying that upstream regulatory components – rather than structural changes in proline‐metabolic enzymes – underlie this transcriptional and metabolic configuration. Thus, WS1 provides a useful system to examine how a water‐saving phenotype can be maintained through metabolic reprogramming in the apparent absence of strong canonical ABA activation.

### 4.3 SnRK1‐Related Phosphorylation Activates Drought‐Associated States not via Canonical ABA Responsiveness

Among phosphopeptides detected exclusively in WS1 or at > 3‐fold higher abundance compared with C (Supporting Information Tables S2 and 3), we identified phosphorylated peptides derived from AKINBETA1 (TraesCS4D03G0448500 and TraesCS4B03G0502600), a β‐subunit of SnRK1, as well as from OXS3 (TraesCS1A03G0558500 and TraesCS1D03G0589400), and from trehalose 6 phosphate phosphatase/synthase (TPS, TraesCS5A03G0527400). In Arabidopsis, SnRK1‐mediated phosphorylation of OXS3 has been linked to ABA‐independent regulation of ABA‐related transcriptional outputs (Xiao et al. 2021). The presence of phosphorylated components consistent with a SnRK1–OXS3–TPS module therefore suggests that WS1 may engage an alternative regulatory configuration that can coincide with drought‐associated traits without requiring strong induction of canonical ABA signalling.

SnRK1 has also been connected to broader metabolic regulation, including sugar signalling and interactions with ABA‐related processes (Jossier et al. 2009; Wurzinger et al. 2018; Carianopol et al. 2020; Belda‐Palazón et al. 2022; Zhao et al. 2026). Moreover, SnRK1‐associated states have been implicated in asparagine‐related metabolism and other adjustments in primary metabolism in plants (Jossier et al. 2009; Wurzinger et al. 2018; Tsai and Gazzarrini 2014; Hayami and Y. Yamamoto 2021). In WS1, transcriptome data indicated sustained upregulation of asparagine‐related metabolism (Supporting Information Figure S4a), consistent with a nitrogen‐associated metabolic configuration under a stress‐associated state. While our data do not establish causality, the combined phosphoproteomic and transcriptomic patterns support the view that WS1 adopts an altered energy‐ and metabolism‐related regulatory state that is compatible with ABA‐independent like expression of water‐saving drought‐associated traits.

### 4.4 BSL3 Phosphorylation Suggests Modulation of Hormone‐Linked Signalling Outputs

We also detected phosphorylated peptides corresponding to BRI1 suppressor 1 (BSU1)‐like 3 (BSL3) (TraesCS5B03G0943200). In Arabidopsis, phosphorylation‐dependent activity of BSL family proteins can influence downstream signalling modules, including crosstalk between brassinosteroid‐ and ABA‐related pathways, for example via regulation of BIN2 activity (Wang et al. 2020b). Although the precise functional meaning of BSL3 phosphorylation in wheat remains to be resolved, it is plausible that BSL3‐associated phosphorylation could contribute to tuning downstream hormone‐linked outputs in WS1, potentially dampening the magnitude of ABA‐responsive transcription despite the presence of drought‐associated traits.

Notably, transcriptome data showed downregulation of steroid biosynthesis‐related genes upstream of brassinosteroid production in WS1, suggesting that BSL3 phosphorylation in this line may not simply reflect canonical BR activation. Instead, these results raise the possibility that phosphorylation‐based regulation contributes to shifting WS1 towards a survival‐oriented state through a mechanism that is at least partly uncoupled from classical hormone biosynthesis and signalling pathways.

### 4.5 Linking SnRK1‐Associated States to Proline Accumulation, Metabolic Reprogramming and Finally Water‐Saving

Although both proline and SnRK1 are implicated in drought responses, the mechanistic relationship between them remains unclear in crops. Our results suggest that proline accumulation in WS1 co‐occurs with phosphorylation patterns consistent with altered SnRK1‐associated regulation. In Arabidopsis, SnRK1 phosphorylates and activates the transcription factor bZIP63, promoting metabolic reprogramming under low‐energy conditions, including induction of ProDH (Mair et al. 2015). In WS1, however, phosphorylated bZIP family proteins were not detected, and *TaProDH1* expression was unchanged, whereas proline biosynthetic genes were upregulated and proline accumulated (Figure 2f–j). These observations suggest that the SnRK1‐associated configuration in WS1 may differ from classical starvation‐type responses and may reflect a distinct, drought‐associated phosphorylation landscape.

Phosphorylation site selection and combinatorial phosphorylation can substantially alter downstream regulatory outputs (Hartmann et al. 2015). Accordingly, the phosphorylation patterns detected in WS1 may reflect a drought‐associated regulatory state that supports metabolic reprogramming towards osmoprotectant accumulation, while avoiding induction of catabolic proline turnover. Although direct phosphorylation‐based activation of P5CS or P5CR by SnRK1 has not been demonstrated, SnRK1 is widely recognised as a central regulator of stress‐associated metabolic adjustment (Polge and Thomas 2007; Wurzinger et al. 2018; Wang et al. 2020a), and proline metabolism is positioned as a regulatory hub across multiple stress contexts (Alvarez et al. 2022; Zhang and Becker 2015). Overexpression of P5CS to induce proline accumulation enhanced osmotic or salt stress tolerance in plants, implying a close relationship between proline accumulation and water‐saving (Kishor et al. 1995; Hmida‐Sayari et al. 2005). Together, these findings support a functional link between SnRK1‐associated regulation and proline‐centred metabolic adjustment in WS1, while emphasising the need for future genetic dissection to establish causal relationships.

### 4.6 Functional Implications of EMS‐Induced Mutations: Candidate Nodes for Future Genetic Dissection

Exome sequencing of WS1 identified 80 genes carrying EMS‐induced nonsense mutations (Krasileva et al. 2017) (Supporting Information Figure S7 and Table S6). Among these, TraesCS1A03G0845300 corresponds to an orthologue of Arabidopsis hexokinase 2 (HXK2), which phosphorylates glucose to glucose‐6‐phosphate (G6P) and influences the hexose phosphate pool. Because hexose phosphates such as G6P and trehalose‐6‐phosphate (T6P) converted by TPS from hexose phosphate pools can inhibit SnRK1 activity (Zhang et al. 2009; Nunes et al. 2013), reduced HXK2 function could, in principle, decrease inhibitory hexose phosphate pools and thereby bias the system towards a SnRK1‐associated state. This provides a potential mechanistic link between altered sugar signalling and the observed phosphorylation state. Consistent with this framework, TPS, HXK and SnRK1 have been highlighted as core regulators of growth–stress balance (Li and Zhao 2024). Although this scenario remains hypothetical at present, it provides a tractable route for future genetic analysis of WS1.

Another nonsense mutation was found in TraesCS6A03G0521700, homologous to Arabidopsis MYB3R‐4, a regulator of cell division (Yang et al. 2021). This could contribute to the developmental phenotypes observed in WS1, including delayed growth and altered spike traits. In line with this possibility, GO enrichment analysis of the WS1 phosphoproteome highlighted cell division‐related categories such as mitotic sister chromatid cohesion, chromosome organisation and cell cycle processes (Figure 5c). Therefore, the reduced fertility in WS1 may reflect, at least in part, altered regulation of cell‐cycle‐associated processes, consistent with a survival‐oriented resource allocation strategy.

### 4.7 Integrative Model: Phosphorylation‐ and Metabolism‐Associated Framework for a Water‐Saving Drought Survival Phenotype

The transcriptomic and phosphoproteomic changes observed in WS1 are likely coordinated components of a common regulatory state, rather than independent processes. Together, integration of transcriptomic, phosphoproteomic and mutational datasets indicates that WS1 expresses a water‐saving drought survival phenotype accompanied by coordinated metabolic and phosphorylation reprogramming (Figure 6). Our data suggest that SnRK1‐related energy signalling and ABA_toggle‐independent stress‐associated transcription contribute to the WS1 phenotype, including components such as OXS3 and TPS (Griffith et al. 2026; Xiao et al. 2021; Wurzinger et al. 2018). Recent work further supports the potential of manipulating sugar–energy signalling pathways to improve stress performance and yield‐related traits in crops (Wurzinger et al. 2018; Li and Zhao 2024; Griffiths et al. 2026). However, no genes related to osmoprotectant proline accumulation were identified in the phosphoproteome or in EMS‐induced mutations from a series of our study. Therefore, osmoprotectant proline accumulation may be regulated independently of SnRK1‐associated pathway in WS1. Because WS1 carries multiple EMS‐induced mutations, causal genetic determinants remain to be established. Nevertheless, this study proposes the feasible genes such as AKINBETA1 (TraesCS4D03G0448500 and TraesCS4B03G0502600), HXK2 (TraesCS1A03G0845300), TPS (TraesCS5A03G0527400) and OXS3 (TraesCS1A03G0558500 and TraesCS1D03G0589400) to regulate the WS1 phenotype.

## 5 Conclusions

Importantly, WS1 also exhibits reduced fertility and yield, underscoring a key trade‐off between survival‐oriented water‐saving strategies and reproductive investment. WS1 appears to adopt a constitutive stress‐responsive state that favours survival under drought at the expense of reproductive investment. Accordingly, WS1 represents a drought‐survival strategy in which resource allocation is biased toward stress tolerance rather than constitutive growth defects. Meanwhile, because WS1 originates from EMS mutagenesis, multiple mutations are likely present, and the causal genetic determinants remain to be identified. Therefore, future genetic dissection using segregating populations and genotyping will be necessary to disentangle causal mutations and to determine whether specific phosphorylation site combinations can be leveraged to optimise water‐saving and drought survival while minimising yield penalties. Nonetheless, the WS1 system provides a valuable framework for linking drought‐associated water saving, metabolic adjustment and phosphorylation states in wheat.

## Conflicts of Interest

The authors declare no conflicts of interest.

## Supporting information

Supporting File 1

Supporting File 2

## Data Availability

LC‐MS/MS raw data of phosphoproteomic analysis have been deposited in Japan Proteome Standard Repository/Database (jPOSTP) (Okuda et al. 2025). Phosphoproteomics; link (https://repository.jpostdb.org/preview/1095877145698b148dda985), Access key (1758). Raw and processed RNA‐seq data were deposited in the National Centre for Biotechnological Information Gene Expression Omnibus (NCBI GEO) under accession number GSE316071. In‐house codes used in this study are available through the GitHub repository (https://github.com/junesk9).

## References

[pce70546-bib-0001] Alvarez, M. E. , A. Savouré , and L. Szabados . 2022. “Proline Metabolism as Regulatory Hub.” Trends in Plant Science 27: 39–55. 34366236 10.1016/j.tplants.2021.07.009

[pce70546-bib-0002] Asseng, S. , F. Ewert , P. Martre , et al. 2015. “Rising Temperatures Reduce Global Wheat Production.” Nature Climate Change 5: 143–147.

[pce70546-bib-0003] Baena‐González, E. , and J. Sheen . 2008. “Convergent Energy and Stress Signaling.” Trends in Plant Science 13: 474–482. 18701338 10.1016/j.tplants.2008.06.006 PMC3075853

[pce70546-bib-0004] Belda‐Palazón, B. , M. Costa , T. Beeckman , F. Rolland , and E. Baena‐González . 2022. “ABA Represses TOR and Root Meristem Activity Through Nuclear Exit of the SnRK1 Kinase.” Proceedings of the National Academy of Sciences 119: e2204862119. 10.1073/pnas.2204862119 PMC9282376 35787039

[pce70546-bib-0005] Bolger, A. M. , M. Lohse , and B. Usadel . 2014. “Trimmomatic: A Flexible Trimmer for Illumina Sequence Data.” Bioinformatics 30: 2114–2120. 24695404 10.1093/bioinformatics/btu170 PMC4103590

[pce70546-bib-0006] Carianopol, C. S. , A. L. Chan , S. Dong , N. J. Provart , S. Lumba , and S. Gazzarrini . 2020. “An Abscisic Acid‐Responsive Protein Interaction Network for Sucrose Non‐Fermenting Related Kinase1 in Abiotic Stress Response.” Communications Biology 3: 145. 32218501 10.1038/s42003-020-0866-8 PMC7099082

[pce70546-bib-0007] Chen, H. H. , L. Qu , Z. H. Xu , J. K. Zhu , and H. W. Xue . 2018. “EL1‐like Casein Kinases Suppress ABA Signaling and Responses by Phosphorylating and Destabilizing the ABA Receptors PYR/PYLs in Arabidopsis.” Molecular Plant 11: 706–719. 29505832 10.1016/j.molp.2018.02.012

[pce70546-bib-0008] Cunningham, F. , J. E. Allen , J. Allen , et al. 2022. “Ensembl 2022.” Nucleic Acids Research 50: D988–D995. 34791404 10.1093/nar/gkab1049 PMC8728283

[pce70546-bib-0009] Cutler, S. R. , P. L. Rodriguez , R. R. Finkelstein , and S. R. Abrams . 2010. “Abscisic Acid: Emergence of a Core Signaling Network.” Annual Review of Plant Biology 61: 651–679. 10.1146/annurev-arplant-042809-112122 20192755

[pce70546-bib-0010] Farquhar, G. D. , J. R. Ehleringer , and K. T. Hubick . 1989. “Carbon Isotope Discrimination and Photosynthesis.” Annual Review of Plant Physiology and Plant Molecular Biology 40: 503–537.

[pce70546-bib-0011] Flavell, R. B. 2023. “A Framework for Improving Wheat Spike Development and Yield Based on the Master Regulatory TOR and SnRK Gene Systems.” Journal of Experimental Botany 74: 755–768. 36477879 10.1093/jxb/erac469 PMC9899413

[pce70546-bib-0012] Griffiths, C. A. , X. Xue , J. A. Miret , et al. 2026. “Membrane‐Permeable Trehalose 6‐phosphate Precursor Spray Increases Wheat Yields in Field Trials.” Nature Biotechnology 44: 316–325. 10.1038/s41587-025-02611-1 PMC12909131 40301657

[pce70546-bib-0013] Gupta, A. , A. Rico‐Medina , and A. I. Caño‐Delgado . 2020. “The Physiology of Plant Responses to Drought.” Science 368: 266–269. 32299946 10.1126/science.aaz7614

[pce70546-bib-0014] Hartmann, J. , D. Linke , C. Bönniger , A. Tholey , and M. Sauter . 2015. “Conserved Phosphorylation Sites in the Activation Loop of the Arabidopsis Phytosulfokine Receptor PSKR1 Differentially Affect Kinase and Receptor Activity.” Biochemical Journal 472: 379–391. 26472115 10.1042/BJ20150147 PMC4661564

[pce70546-bib-0015] Hayami, N. , and Y. Y. Yamamoto . 2021. “Primary Metabolism and Transcriptional Regulation in Higher Plants.” Reviews in Agricultural Science 9: 117–127.

[pce70546-bib-0016] Heino, M. , P. Kinnunen , W. Anderson , et al. 2023. “Increased Probability of Hot and Dry Weather Extremes During the Growing Season Threatens Global Crop Yields.” Scientific Reports 13: 3583. 36869041 10.1038/s41598-023-29378-2 PMC9984494

[pce70546-bib-0017] Hmida‐Sayari, A. , R. Gargouri‐Bouzid , A. Bidani , L. Jaoua , A. Savouré , and S. Jaoua . 2005. “Overexpression of Δ^1^‐pyrroline‐5‐carboxylate Synthetase Increases Proline Production and Confers Salt Tolerance in Transgenic Potato Plants.” Plant Science 169: 746–752.

[pce70546-bib-0018] Itam, M. , R. Mega , S. Tadano , et al. 2020. “Metabolic and Physiological Responses to Progressive Drought Stress in Bread Wheat.” Scientific Reports 10: 17189. 33057205 10.1038/s41598-020-74303-6 PMC7560863

[pce70546-bib-0019] Jossier, M. , J. P. Bouly , P. Meimoun , et al. 2009. “SnRK1 (SNF1‐related Kinase 1) has a Central Role in Sugar and ABA Signalling in *Arabidopsis thaliana* .” Plant Journal 59: 316–328. 10.1111/j.1365-313X.2009.03871.x 19302419

[pce70546-bib-0020] Kim, J. S. , M. Sato , M. Kojima , et al. 2025. “Multiomics‐Based Assessment of the Impact of Airflow on Diverse Plant Callus Cultures.” Scientific Data 12: 197. 39900939 10.1038/s41597-025-04518-7 PMC11790825

[pce70546-bib-0021] Kishor, P. , Z. Hong , G. H. Miao , C. Hu , and D. Verma . 1995. “Overexpression of [delta]‐Pyrroline‐5‐Carboxylate Synthetase Increases Proline Production and Confers Osmotolerance in Transgenic Plants.” Plant Physiology 108: 1387–1394. 12228549 10.1104/pp.108.4.1387 PMC157516

[pce70546-bib-0022] Krasileva, K. V. , H. A. Vasquez‐Gross , T. Howell , et al. 2017. “Uncovering Hidden Variation in Polyploid Wheat.” Proceedings of the National Academy of Sciences, USA 115: E913–E921. 10.1073/pnas.1619268114 PMC5307431 28096351

[pce70546-bib-0023] Lea, P. J. , L. Sodek , M. A. J. Parry , P. R. Shewry , and N. G. Halford . 2007. “Asparagine in Plants.” Annals of Applied Biology 150: 1–26.

[pce70546-bib-0024] Lesk, C. , P. Rowhani , and N. Ramankutty . 2016. “Influence of Extreme Weather Disasters on Global Crop Production.” Nature 529: 84–87. 26738594 10.1038/nature16467

[pce70546-bib-0025] Li, G. , and Y. Zhao . 2024. “The Critical Roles of Three Sugar‐Related Proteins (HXK, SnRK1, TOR) in Regulating Plant Growth and Stress Responses.” Horticulture Research 11: uhae099. 38863993 10.1093/hr/uhae099 PMC11165164

[pce70546-bib-0026] Li, X. , X. Kong , Q. Huang , et al. 2019. “CARK1 Phosphorylates Subfamily III Members of ABA Receptors.” Journal of Experimental Botany 70: 519–528. 30380101 10.1093/jxb/ery374 PMC6322571

[pce70546-bib-0027] Lobell, D. B. , W. Schlenker , and J. Costa‐Roberts . 2011. “Climate Trends and Global Crop Production Since 1980.” Science 333: 616–620. 21551030 10.1126/science.1204531

[pce70546-bib-0028] Love, M. I. , W. Huber , and S. Anders . 2014. “Moderated Estimation of Fold Change and Dispersion for RNA‐Seq Data With Deseq. 2.” Genome Biology 15: 550. 25516281 10.1186/s13059-014-0550-8 PMC4302049

[pce70546-bib-0029] Mair, A. , L. Pedrotti , B. Wurzinger , et al. 2015. “SnRK1‐triggered Switch of bZIP63 Dimerization Mediates the Low‐Energy Response in Plants.” eLife 4: e05828. 26263501 10.7554/eLife.05828 PMC4558565

[pce70546-bib-0030] Mega, R. , F. Abe , J. S. Kim , et al. 2019. “Tuning Water‐Use Efficiency and Drought Tolerance in Wheat Using Abscisic Acid Receptors.” Nature Plants 5: 153–159. 30737511 10.1038/s41477-019-0361-8

[pce70546-bib-0031] Nunes, C. , L. F. Primavesi , M. K. Patel , et al. 2013. “Inhibition of SnRK1 by Metabolites: Tissue‐Dependent Effects and Cooperative Inhibition by Glucose 1‐phosphate in Combination With Trehalose 6‐Phosphate.” Plant Physiology and Biochemistry: PPB 63: 89–98. 23257075 10.1016/j.plaphy.2012.11.011

[pce70546-bib-0032] O'Shea, J. P. , M. F. Chou , S. A. Quader , J. K. Ryan , G. M. Church , and D. Schwartz . 2013. “pLogo: A Probabilistic Approach to Visualizing Sequence Motifs.” Nature Methods 10: 1211–1212. 24097270 10.1038/nmeth.2646

[pce70546-bib-0033] Okuda, S. , A. C. Yoshizawa , D. Kobayashi , et al. 2025. “jPOST Environment Accelerates the Reuse and Reanalysis of Public Proteome Mass Spectrometry Data.” Nucleic Acids Research 53: D462–D467. 39526391 10.1093/nar/gkae1032 PMC11701591

[pce70546-bib-0034] Park, S. Y. , P. Fung , N. Nishimura , et al. 2009. “Abscisic Acid Inhibits Type 2C Protein Phosphatases via the PYR/PYL Family of START Proteins.” Science 324: 1068–1071. 19407142 10.1126/science.1173041 PMC2827199

[pce70546-bib-0035] Polge, C. , and M. Thomas . 2007. “SNF1/AMPK/SnRK1 Kinases, Global Regulators at the Heart of Energy Control?” Trends in Plant Science 12: 20–28. 17166759 10.1016/j.tplants.2006.11.005

[pce70546-bib-0036] R Core Team ., R: A Language and Environment for Statistical Computing. https://www.R-project.org/ (2023). https://www.R-project.org/.

[pce70546-bib-0057] Rakszegi, M. , B. N. Kisgyorgy , and K. Tearall , et al. 2010. “Diversity of agronomic and morphological traits in a mutant population of bread wheat studied in the Healthgrain program” Euphytica 174: 409–421.

[pce70546-bib-0037] Rosenzweig, C. , J. Elliott , D. Deryng , et al. 2014. “Assessing Agricultural Risks of Climate Change in the 21st Century in a Global Gridded Crop Model Intercomparison.” Proceedings of the National Academy of Sciences 111: 3268–3273. 10.1073/pnas.1222463110 PMC3948251 24344314

[pce70546-bib-0038] Soneson, C. , M. I. Love , and M. D. Robinson . 2015. “Differential Analyses for RNA‐Seq: Transcript‐Level Estimates Improve Gene‐Level Inferences.” F1000Research 4: 1521. 26925227 10.12688/f1000research.7563.1 PMC4712774

[pce70546-bib-0039] Srivastava, A. , L. Malik , H. Sarkar , et al. 2020. “Alignment and Mapping Methodology Influence Transcript Abundance Estimation.” Genome Biology 21: 239. 32894187 10.1186/s13059-020-02151-8 PMC7487471

[pce70546-bib-0040] Sugiyama, N. , T. Masuda , K. Shinoda , A. Nakamura , M. Tomita , and Y. Ishihama . 2007. “Phosphopeptide Enrichment by Aliphatic Hydroxy Acid‐Modified Metal Oxide Chromatography for Nano‐LC‐MS/MS in Proteomics Applications.” Molecular and cellular proteomics: MCP 6: 1103–1109. 17322306 10.1074/mcp.T600060-MCP200

[pce70546-bib-0041] Tsai, A. Y. L. , and S. Gazzarrini . 2014. “Trehalose‐6‐phosphate and SnRK1 Kinases in Plant Development and Signaling: The Emerging Picture.” Frontiers in Plant Science 5: 1–11. 10.3389/fpls.2014.00119 PMC3978363 24744765

[pce70546-bib-0042] Wang, P. , Y. Zhao , Z. Li , et al. 2018. “Reciprocal Regulation of the TOR Kinase and ABA Receptor Balances Plant Growth and Stress Response.” Molecular Cell 69: 100–112.e6. 29290610 10.1016/j.molcel.2017.12.002 PMC5772982

[pce70546-bib-0043] Wang, Q. , F. Yu , and Q. Xie . 2020b. “Balancing Growth and Adaptation to Stress: Crosstalk Between Brassinosteroid and Abscisic Acid Signaling.” Plant, Cell and Environment 43: 2325–2335. 10.1111/pce.13846 32671865

[pce70546-bib-0044] Wang, Y. , L. Wang , B. J. Micallef , et al. 2020a. “AKINβ1, a Subunit of SnRK1, Regulates Organic Acid Metabolism and Acts as a Global Modulator of Genes Involved in Carbon, Lipid, and Nitrogen Metabolism.” Journal of Experimental Botany 71: 1010–1028. 31624846 10.1093/jxb/erz460

[pce70546-bib-0045] Wurzinger, B. , E. Nukarinen , T. Nägele , W. Weckwerth , and M. Teige . 2018. “The SnRK1 Kinase as Central Mediator of Energy Signaling Between Different Organelles.” Plant Physiology 176: 1085–1094. 29311271 10.1104/pp.17.01404 PMC5813556

[pce70546-bib-0046] Xiao, S. , L. Jiang , C. Wang , and D. W. Ow . 2021. “Arabidopsis OXS3 Family Proteins Repress ABA Signaling Through Interactions With AFP1 in the Regulation of ABI4 Expression.” Journal of Experimental Botany 72: 5721–5734. 34037750 10.1093/jxb/erab237

[pce70546-bib-0047] Yamashita, K. , S. Katagiri , H. Takase , et al. 2025. “MAP4K1 and MAP4K2 Regulate ABA‐Induced and Ca^2+^‐Mediated Stomatal Closure in *Arabidopsis* .” Science Advances 11: eadt4916. 41417897 10.1126/sciadv.adt4916 PMC12716388

[pce70546-bib-0048] Yang, W. , S. Cortijo , N. Korsbo , et al. 2021. “Molecular Mechanism of Cytokinin‐Activated Cell Division in Arabidopsis.” Science 371: 1350–1355. 33632892 10.1126/science.abe2305 PMC8166333

[pce70546-bib-0049] Yoshida, T. , A. Christmann , K. Yamaguchi‐Shinozaki , E. Grill , and A. R. Fernie . 2019. “Revisiting the Basal Role of ABA – Roles Outside of Stress.” Trends in Plant Science 24: 625–635. 31153771 10.1016/j.tplants.2019.04.008

[pce70546-bib-0050] Zhang, L. , and D. F. Becker . 2015. “Connecting Proline Metabolism and Signaling Pathways in Plant Senescence.” Frontiers in Plant Science 6: 552. 26347750 10.3389/fpls.2015.00552 PMC4544304

[pce70546-bib-0051] Zhang, L. , X. Li , D. Li , et al. 2018. “CARK1 Mediates ABA Signaling by Phosphorylation of ABA Receptors.” Cell Discovery 4: 30. 29928509 10.1038/s41421-018-0029-y PMC6006248

[pce70546-bib-0052] Zhang, M. , D. Lv , P. Ge , et al. 2014. “Phosphoproteome Analysis Reveals New Drought Response and Defense Mechanisms of Seedling Leaves in Bread Wheat (*Triticum aestivum* L.).” Journal of Proteomics 109: 290–308. 25065648 10.1016/j.jprot.2014.07.010

[pce70546-bib-0053] Zhang, Y. , L. F. Primavesi , D. Jhurreea , et al. 2009. “Inhibition of SNF1‐Related Protein Kinase1 Activity and Regulation of Metabolic Pathways by Trehalose‐6‐Phosphate.” Plant Physiology 149: 1860–1871. 19193861 10.1104/pp.108.133934 PMC2663748

[pce70546-bib-0054] Zhao, Y. , Z. Wu , J. Zhang , and G. Li . 2026. “Crosstalk and Coordination of TOR, SnRK1, and ABA in Plant Metabolic and Environmental Adaptation.” Plant Science 364: 112965. 41461324 10.1016/j.plantsci.2025.112965

[pce70546-bib-0055] Zhu, T. , L. Wang , H. Rimbert , et al. 2021. “Optical Maps Refine the Bread Wheat Triticum aestivum cv. Chinese Spring Genome Assembly.” Plant Journal 107: 303–314. 10.1111/tpj.15289 PMC8360199 33893684

