# Supplementary material for: A Water‐Saving Drought Survival Phenotype in a Wheat TILLING Mutant Involves Survival‐Biased Metabolic and Phosphorylation Reprogramming
Source: Plant Cell Environ. 2026 Apr 19;49(8):5435–49. doi: 10.1111/pce.70546 (PMC13353719; doi:10.1111/pce.70546)
Supplement: Supplementary file 1 — Supporting File 1 [file PCE-49-5435-s001.pdf]

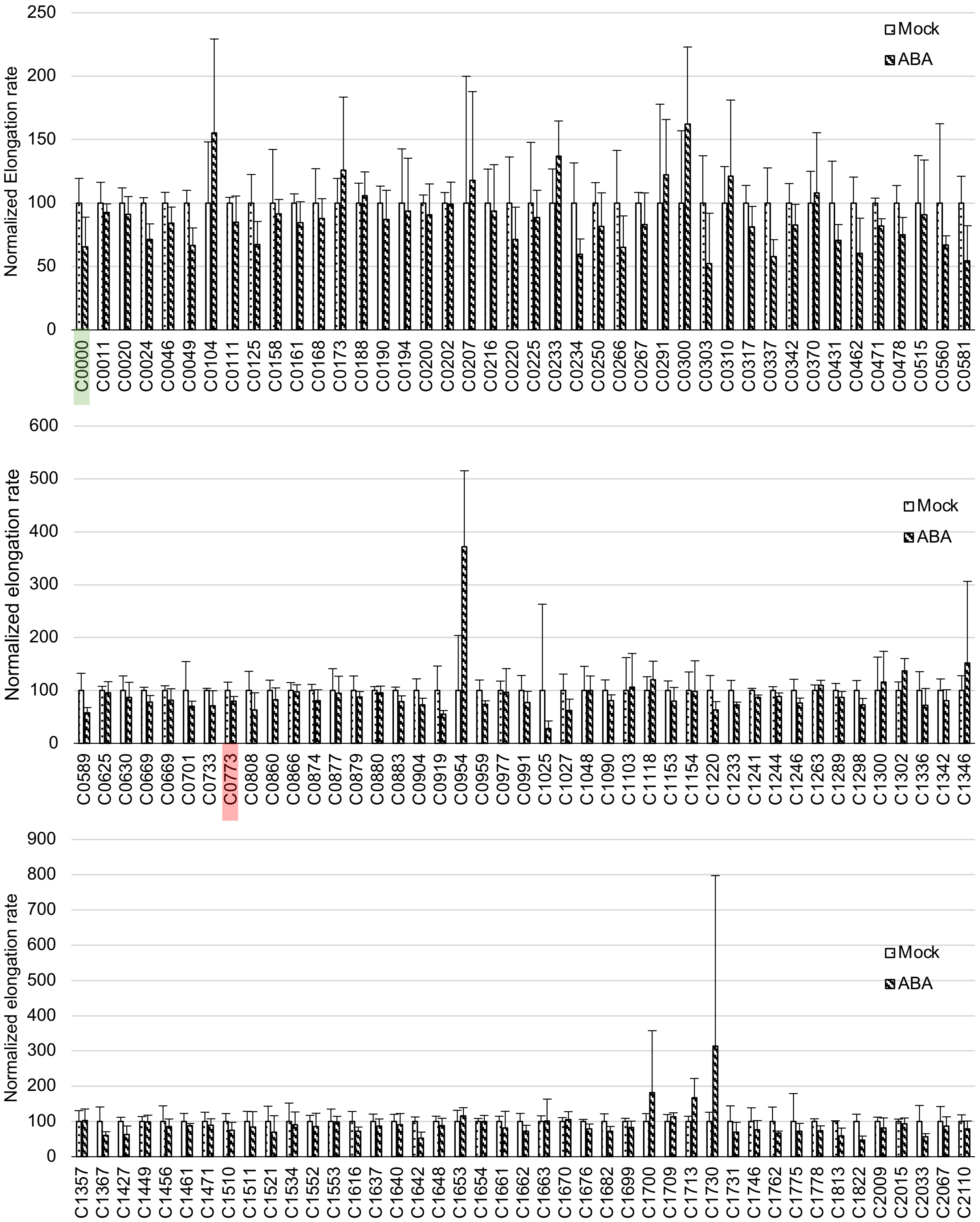

Supplementary Figure 1

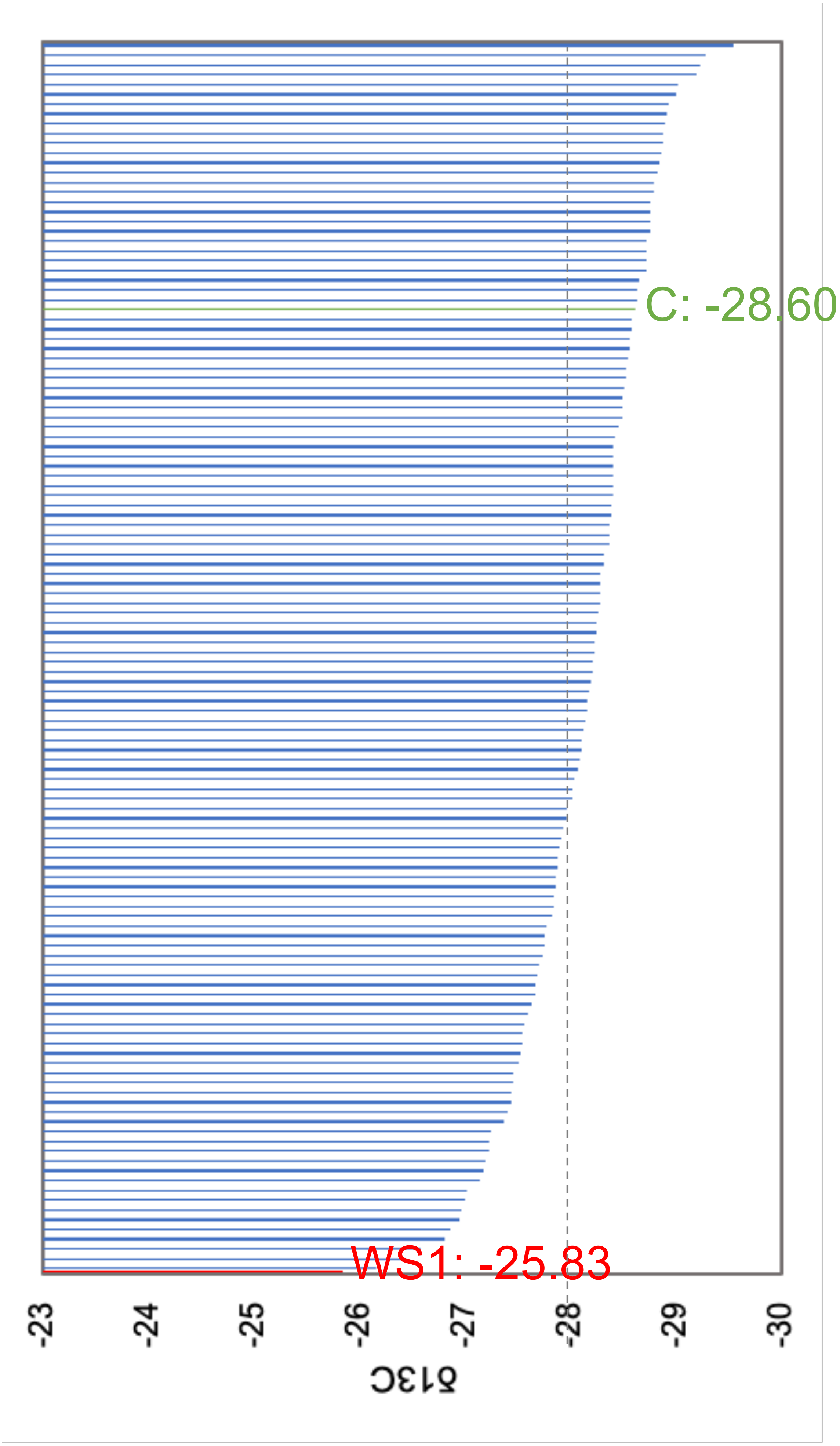

Supplementary Figure 2

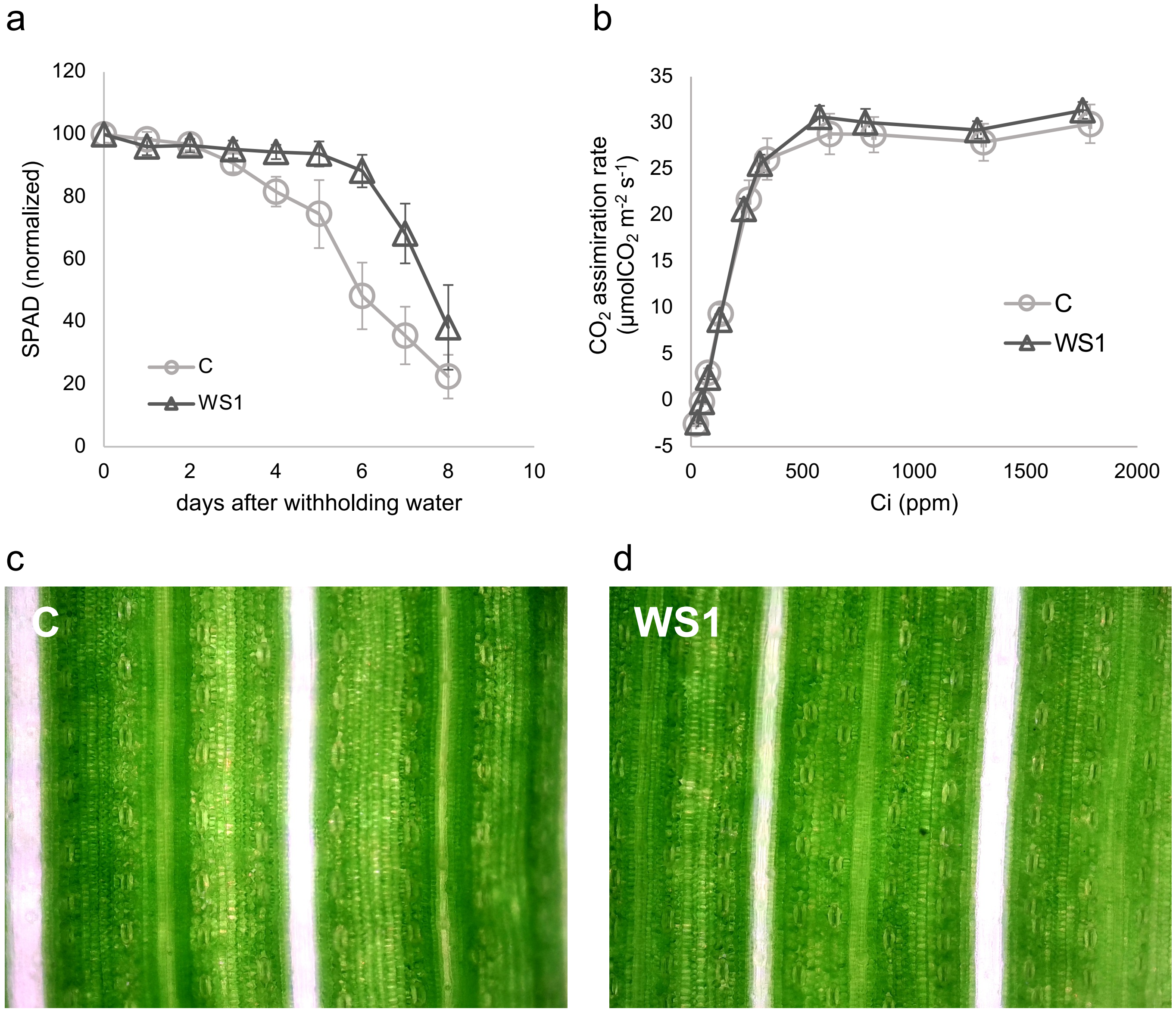

Supplementary Figure 3

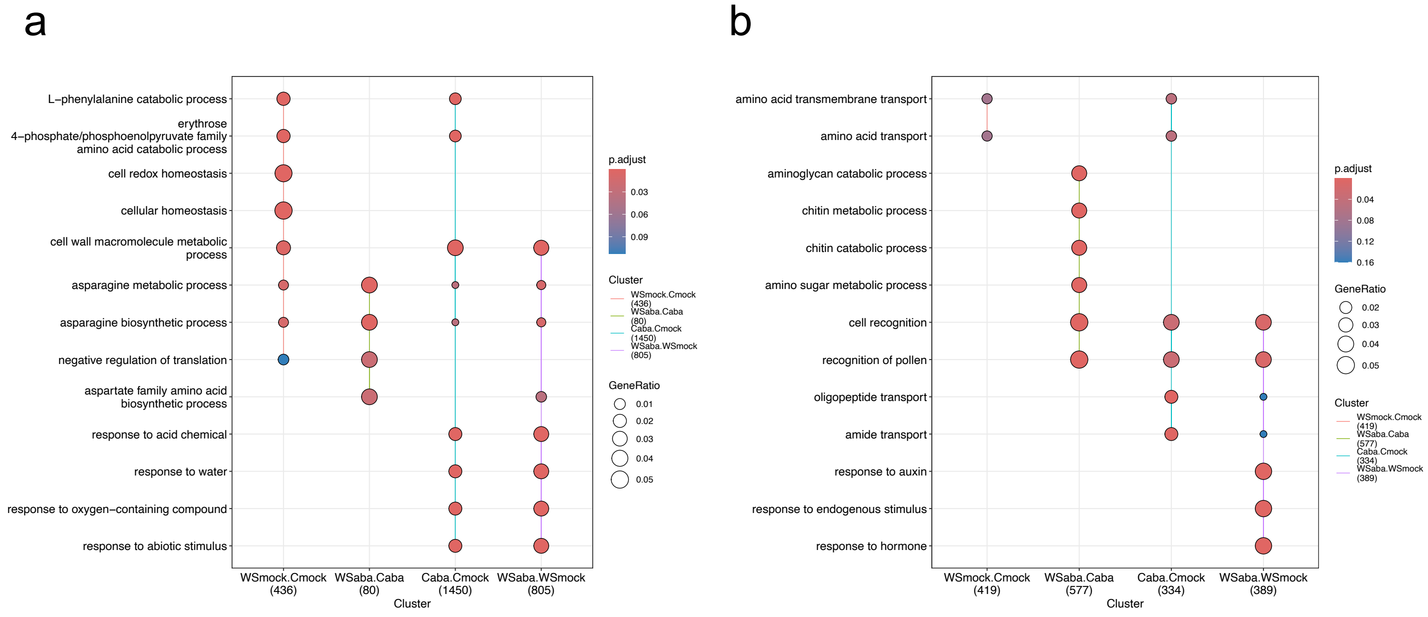

Supplementary Figure 4

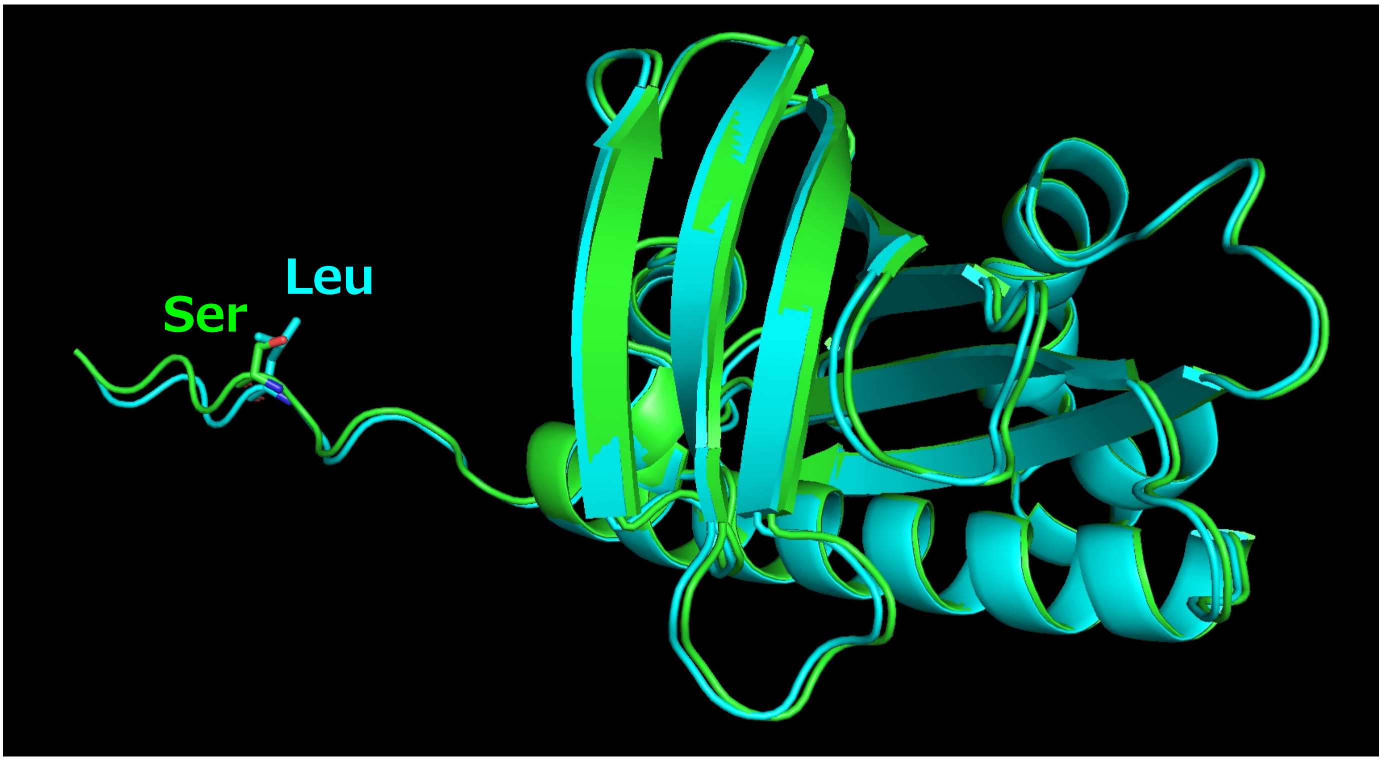

Supplementary Figure 5

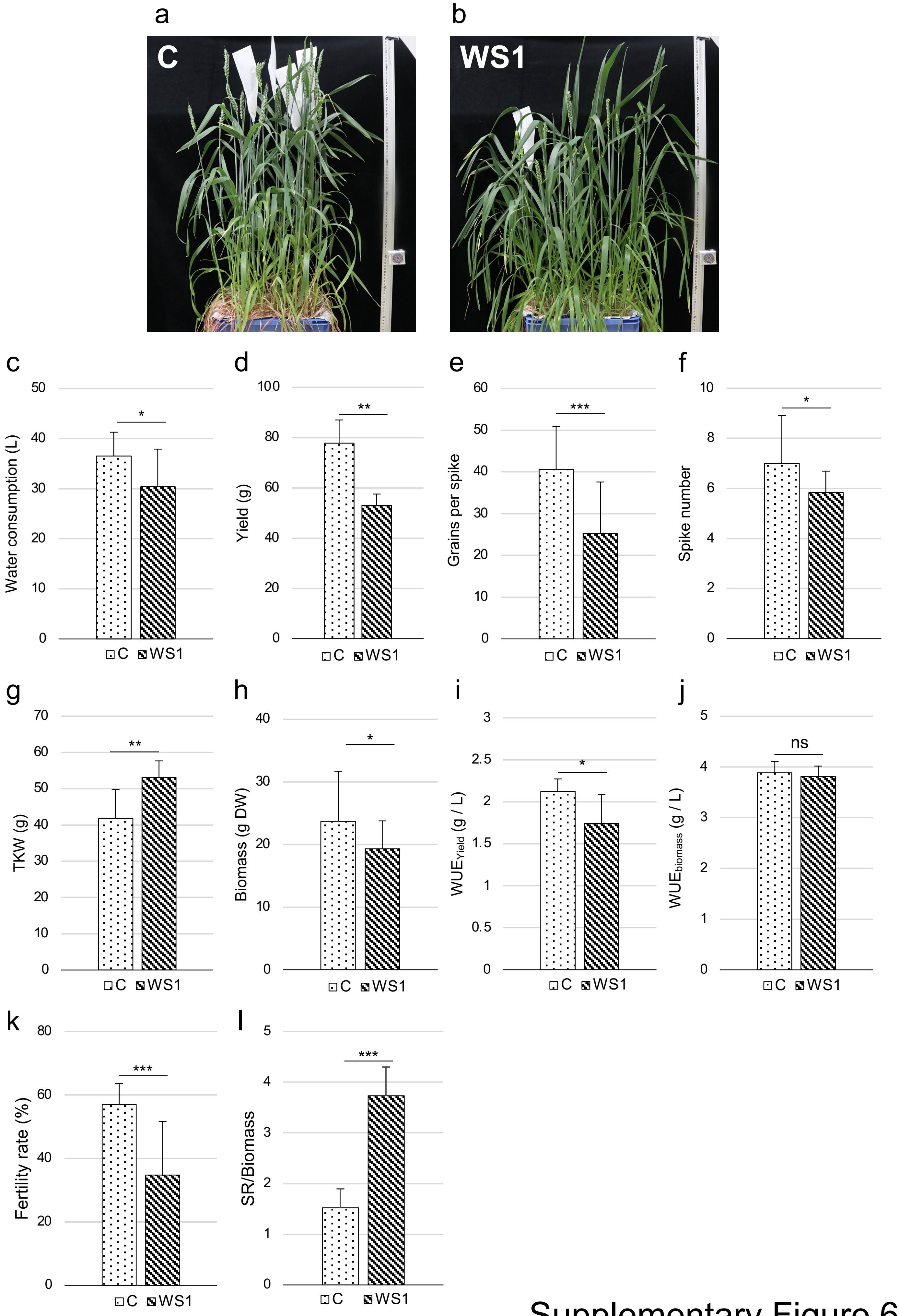

Supplementary Figure 6

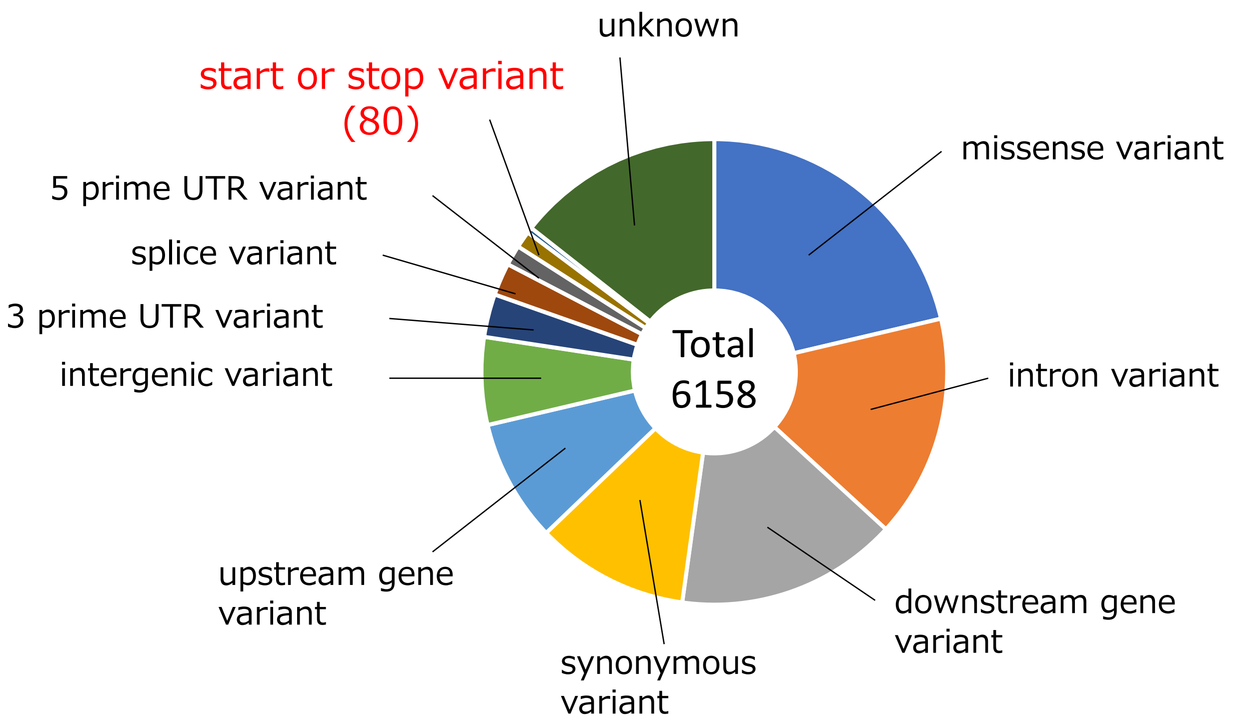

Supplementary Figure 7
